# Reply to Edelstein, “Does *Legionella* 5S rRNA PCR melting temperature analysis discriminate high-risk species and increased disease severity or does it simply reflect test selection bias?”

**DOI:** 10.1128/jcm.01077-26

**Published:** 2026-10-27

**Authors:** Aaron M. Pulsipher, Georges Khattar, Robin Patel, Patricia J. Simner

**Affiliations:** 1 Department of Critical Care, Mayo Clinic 23387 , Phoenix, Arizona, USA; 2 Division of Clinical Microbiology, Mayo Clinic 4352 , Rochester, Minnesota, USA; 3 Division of Public Health, Infectious Disease, and Occupational Medicine, Mayo Clinic 1466 , Rochester, Minnesota, USA; Division of Intramural Research, National Institute of Allergy and Infectious Diseases, Bethesda, Maryland, USA

## REPLY

We thank Dr. Edelstein for the thoughtful comments. We note that the fluorescence resonance energy transfer (FRET)-based real-time PCR assay studied was designed for genus-level *Legionella* detection, not to identify specific species; species have not been reported from the PCR assay in our clinical practice. During assay validation, *L. pneumophila* and *L. longbeachae* demonstrated melting temperature (Tm) values distinct from those of other *Legionella* species, enabling assay-specific group-level species discrimination, as further supported by the real-world culture comparison presented (1). Accordingly, in the study, Tm was used to distinguish *L. pneumophila* (Tm ≥ 66°C) from lower Tm (<66°C) non-*pneumophila* species, with the exception of *L. longbeachae. L. longbeachae* was grouped with *L. pneumophila* because of assay characteristics (Tm ≥ 66°C). We agree that specific conclusions about *L. longbeachae* are limited based on the data presented.

Although non-*pneumophila Legionella* species have historically been considered to primarily affect immunocompromised hosts, the findings presented suggest that immunocompetent patients may be affected by non-*pneumophila Legionella* species as well; immunocompetent patients comprised similar proportions of the Tm < 66°C and Tm ≥ 66°C groups (42.9 and 38.3%, respectively; *P* = 0.63).

We agree that differential diagnostic ascertainment bias is an important limitation of this retrospective *Legionella* cohort; diagnostic testing practices influence all real-world infection studies. Decisions to pursue *Legionella* testing rest with healthcare providers informed by current recommendations, which encourage testing of patients with severe community-acquired pneumonia (CAP) and immunocompromised patients with pneumonia (2, 3). Consequently, immunocompromised patients with milder disease may be more likely to undergo diagnostic evaluation than immunocompetent patients with similar illness. At our institution, *Legionella* PCR is incorporated into an immunocompromised host bronchoscopy testing algorithm. In our published multicenter cohort of patients with *Legionella* pneumonia (4), 71.6% of those who were immunocompromised had undergone bronchoscopy *versus* 33.9% of those who were immunocompetent, increasing opportunities for lower respiratory tract PCR-based diagnosis and detection of non-*Legionella pneumophila* species and contributing to differential ascertainment.

To assess diagnostic timing, the interval from hospital presentation to the first *Legionella* test (urine antigen, PCR, or culture) among hospitalized patients was evaluated. Median time to the first diagnostic test collection was similar between the Tm < 66°C and Tm ≥ 66°C groups (22.5 *versus* 17.9 h, respectively; *P* = 0.37), suggesting comparable early clinical suspicion. PCR specimen collection, however, occurred later in the former group (68.0 *versus* 48.5 h, respectively; *P* = 0.007). This difference may reflect variation in respiratory specimen acquisition timing, rather than delayed consideration of *Legionella* infection, given that time to initial *Legionella* testing was similar. Unfortunately, our retrospective cohort does not permit assessment of the frequency of *Legionella* testing among immunocompromised and immunocompetent patients because it lacks the denominator of all patients evaluated for CAP.

We agree that the observed association may reflect true species-associated differences, differential diagnostic ascertainment, or both. Definitive distinction between these possibilities would require a prospective, systematic *Legionella* testing of consecutive patients meeting predefined criteria for CAP. Regardless of the contribution of ascertainment bias, the findings presented demonstrate that broad-range genus-level *Legionella* PCR identifies clinically important non-*pneumophila* infections that would not be detected by urinary antigen testing or assays targeting only *L. pneumophila*.
